# Uncomfortable People

**Published:** 1893-03

**Authors:** 


### UNCOMFORTABLE PEOPLE.

"Yes, my dear, it is very nice indeed, but don't you think it would have been better if you had made it so?" was the stereotyped remark of a woman otherwise very amiable, intelligent and pleasant to have around, says an observant writer. She had unusual ability, was capital as an advisor in all emergencies, met every condition in life with practical philosophy that smoothed out all obstructions, but actually poisoned the entire pleasure of her acquaintance with that everlasting. "Don't you think it would have been better if you had done it some other way?"

There are few things in the world more exasperating than the constant nagging of people who think their way is the best and have no hesitation in informing their friends of their belief.

Everybody has ideas and ways of his own and it would be, indeed, a monotonous world if every people, community or family followed the taste or judgment of some one individual.

Circumstances sometimes seem to have set up an arbiter in a certain locality, and if this leading light happens to be of the arrogant and self-assertive description, the last state of that neighborhood is worse than the first. The best one may do, the best one may think, the choicest articles one may select are scanned with a critical, although possibly pleasant and benevolent eye, but like the tail to a comet comes the expression: "But don't you think it would have been better so?" and in these cases, the tail, like that of the comet, is a good deal larger than all the rest of the situation.

All the pleasure is taken out of life by these people. Good intentions go for naught beyond a certain point, and when all is said and done, and the best is offered, there is an uncomfortable feeling that one is a sort of unprofitable servant and there is something wrong somewhere.

These people should be colonized in a community by themselves and should be so situated as to be forced to take some doses of their own medicine. They might, after a long and severe course of this sort of treatment, come to discover that there are persons in the world who have ideas as well as themselves, and that possibly, only just possibly, of course, these ideas may be quite as good as their own.—*Ex.*

